# Invasiveness of mouse embryos to human ovarian cancer cells HO8910PM and the role of MMP-9

**DOI:** 10.1186/1475-2867-12-23

**Published:** 2012-06-06

**Authors:** Xiaoyan Ding, Liaoqiong Fang, Hong Zhang, Hai Qiao, Zhi-biao Wang

**Affiliations:** 1 State Key Laboratory of Ultrasound Engineering in Medicine Co-founded by Chongqing and the Ministry of Science and Technology, Chongqing Key laboratory of Ultrasound in Medicine and Engineering, College of Biomedical Engineering, Chongqing Medical University, Chongqing, 400016, China

**Keywords:** Mouse embryos, Ovarian cancer, Co-culture, MMP-9

## Abstract

**Background:**

Our previous work found that mouse embryos could invade malignant cancer cells. In the process of implantation, embryo trophoblast cells express matrix metalloproteinases and the invasive ability of trophoblast cells is proportional to matrix metalloproteinase-9 protein expression. So the purpose of this study is to observe the effects of mouse embryos on human ovarian cancer cells in the co-culture environment in vitro and explore the possible mechanism of matrix metalloproteinase-9.

**Methods:**

Several groups of human ovarian cancer cells HO8910PM were co-cultured with mouse embryos for different time duration, after which the effects of mouse embryos on morphology and growth behavior of HO8910PM were observed under the light microscope real-time or by H.E staining. Apoptosis was detected under laser confocal microscope by Annexin V-EGFP/PI staining in situ. Invasion ability of tumor cells was studied by transwell experiments. After matrix metalloproteinase 9 (MMP −9) activity was inhibited by MMP-9 Inhibitor I, the interaction between mouse embryos and human ovarian cancer cells HO8910PM was observed.

**Results:**

Mouse embryos were able to invade co-cultured human ovarian cancer cell layer which extended in the bottom of the culture dish, and gradually pushed away tumor cells to form their own growth space. The number of apoptosis tumor cells surrounding the embryo increased under laser confocal microscope. After co-cultured with mouse embryos, tumor cells invasive ability was lowered compared with the control group. After MMP-9 activity was inhibited, the interaction between mouse embryos and HO8910PM cells had no significant difference compared with the normal MMP-9 activity group.

**Conclusion:**

Mouse embryos were able to invade human ovarian cancer cells *in vitro* and form their own growth space, promote apoptosis of human ovarian cancer cells and lower their invasive ability. The mouse embryo was still able to invade human ovarian cancer cells after MMP-9 activity was inhibited.

## Introduction

Ovarian cancer is one of the most common female genital cancers and its incidence rate ranks only second to cervical cancer and uterine cancer while its fatality rate is in the first place among various types of gynecologic cancers [1]. Its 5-year survival rate is just 30% and this figure has not changed for the past 30 years [2]. Apoptosis, also known as shrinkage necrosis, programmed cell death or cell suicide, plays an important role in removing aging and abnormal cells of the body and maintaining many cell functions [3]. In tumor cells, anti-apoptotic mechanism often abnormally increases, while the pro-apoptotic mechanism hinders which makes tumor cells escape apoptosis. Therefore, if certain method to increase apoptosis of tumor cells can be found, it will contribute to the clinical treatment of cancer [4,5].

The similarity of early embryos and malignant cells attracts the attention of scientists. Murray and his colleagues demonstrated that early embryos and malignant cells shared significant similarity on proliferation, division, invasion, immune escape and angiogenesis, etc [6]. However, in our previous research we found that mouse embryos widely invaded malignant cells which seemingly implied that early embryos had more invasive ability than malignant cells [7]. Although we have done lots of studies about the mechanism of this phenomenon, the exact molecular pathway is still elusive.

Matrix metalloproteinases (MMPs) are a big family, named for their need for Ca, Zn and other metal ions as a cofactor, which can degrade extracellular matrix components and play a key role in the process of tumor invasion and metastasis [8] and angiogenesis [9]. At present in rectal cancer [10], bladder cancer [11], cervical cancer [12], ovarian cancer [13] and other tumors, MMP-9 was found to be involved in the invasion and metastasis of cancer cells and tumor angiogenesis. In the process of implantation embryo trophoblast cells also express MMPs and the invasive ability of trophoblast is proportional to MMP-9 protein expression [14]. Yudate [15] and other studies have found that the expression of MMP-2 and MMP-9 in the first trimester trophoblast cells was even higher than that in choriocarcinoma cell lines BeWo, and concluded that trophoblast cells showed a higher invasion behavior.

Based on the previous work and the co-cultured model of mouse embryos and human ovarian cancer cells, we take into account that in the co-culture environment whether the invasion behavior of ovarian cancer cells is in relation with MMP-9 expression. In this study, by using MMPI to inhibit MMP-9 activity, the role of MMP-9 in the process of embryos invading tumor cells in vitro was explored.

## Materials and methods

### Cell culture

Human ovarian cancer cells HO8910PM were purchased from Chinese Type Culture Collection in WuHan University (WuHan, China). Cell lines were maintained in RPMI 1640 medium (Gibco, New York, USA) containing 10% fetal bovine serum (Gibco, New York, USA) in a humidified atmosphere of 5% CO2 at 37°C. When co-cultured with mouse pre-implantation embryos, cells were fed in Dulbecco’s modified Eagle’medium/F12 (D-MEM/F-12) (Gibco, New York, USA) containing 20% fetal bovine serum.

### Mouse blastocyst acquisition

C57BL/6 mice from Chongqing Medical University experimental animal center were housed at 25°C environment under a 12:12 h light/dark cycle. Food and water were available feeding libitum at all times. Four-to six-week-old female mice were superovulated by an i.p. injection of 7.5 IU Pregnant Mare Serum Gonadotrophin (PMSG)(Everest Biotech Ltd., Oxfordshire, UK) per mouse followed by a second i.p. injection of 7.5 IU Human Chorionic Gonadotropin(HCG) (Everest Biotech Ltd., Oxfordshire, UK) 48 h later. Then the females were mated with six-to eight-week-old male mice. In the next morning, the female mice with vaginal plug were separated from male mice, deemed to be Day 1 of pregnancy (1dpc, postcoitum) and killed on Day 4 to obtain embryos. The use of animals in these procedures was approved by the University Animals Experimental Committee.

### Observation of morphology of interface of cancer cells and embryos

The obtained mouse embryos were set on the layer of prepared cancer cells in the 24-well culture plate, 5 embryos for each well. Then the culture plate was placed in 5% CO2 incubator (SHELLAB TC2323, USA). Morphology and interactions of mouse embryos and cancer cells in co-culture system were observed by the inverted microscopy every 24 hours. After co-cultured with mouse embryos for 48 h, 72 h, 96 h, 120 h, and 144 h, the cancer cells were fixed with paraformaldehyde and Hematoxylin and Eosin (H&E) staining was carried out following the laboratory routine method.

### Apoptosis of cancer cells assay

After human ovarian cancer HO-8910 PM cells were co-cultured with mouse embryos for 48 h, 72 h, 96 h, 120 h in a humidified incubator at 37°C, 5% CO2, they were washed with PBS twice and stained with Annexin V/EGFP-PI Apoptosis Detection Kit(KeyGEN, Nanjing, China) according to the manufacturer's instructions. In brief, 5 ul Annexin V-EGFP and 5 ul propidium iodide were diluted with 500 ul binding buffer and then the mixture was added to the samples for 5 min in the dark. The samples were analyzed under a confocal laser scanning microscopy (LSCM) (Leica TCS-SP2, Solms, Germany).

### Invasion ability measurement

After co-cultured with mouse embryos for 120 hours, tumor cells were digested, centrifugated, then resuspended in serum-free medium, and counted with counting chamber under microscopy to adjust the cell density of 5 × l0^5^/ml. Cell suspension of 200 ul was seeded in the upper layer of Transwell chambers (Coster, USA), under the chamber containing 10% FBS completed medium as chemokines. After culture for 24 h, the polycarbonate membrane was removed and the matrigel gel on it was gently wiped with a cotton swab. Then the membrane was formalin-fixed for 20 minutes and routine hematoxylin eosin (H. E.) stained. Under low magnification microscope, five horizons (up, down, left, right, center) were taken on each membrane. The number of cells which went through the polycarbonate membrane was counted and averaged for each field of vision to indicate the invasive ability of tumor cells. Human ovarian cancer cells without co-culturing were as the control group.

### Inhibition of MMP-9 activity

After co-cultured for 24 hours 0.5 ul of MMP-9 Inhibitor I (Galbiochem, USA) working solution was added to the experimental group, meanwhile 0.5 ul of DMSO was added in the control group (volume of MMP-9 Inhibitor I working solution was based on the manufacture’ instructions, IC_50_ = 5 nM). Both groups were cultured in the 37°C, 5% CO2, relative humidity of 95% -100% incubator. The culture medium was exchanged every 24 h and MMP-9 Inhibitor I working solution and DMSO were added in the experimental group and control group separately. The interaction between embryos and tumor cells was observed under the microscope every day.

## Results

**Figure 1:**
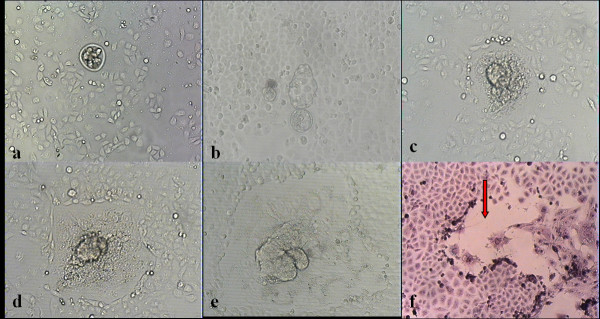
**In co-culture system the embryonic development and its effects on tumor cells in different time stages. (a)** A mouse blastocyst was put on tumor cell layer immediately. **(b)** At 24 h the mouse blastocyst zona pellucida was taking off; **(c)** At 48 h embryonic trophoblast cells adhered and began to expand; **(d)** At 72 h tumor cells were pushed away by the growth of embryos; **(e)** At 120 h the expansion area of trophoblast cells stopped enlarging. (100×) **(f)** A blank gap (red arrow) was seen between mouse embryos and tumor cells. (H&E staining, 200×).

**Figure 2:**
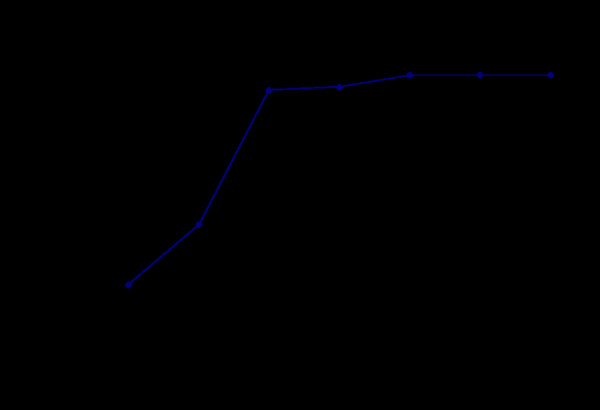
Different outgrowth rate of embryos when co-cultured with HO-8910 PM.

After co-cultured with HO-8910 PM for 24 h, mouse blastocysts hatched from zona pellucida (Figure 1 b). After 48 h, mouse blastocysts attached to the bottoms of cell culture plates which were covered with HO8910PM (Figure 1 c), and the trophoblast cells began to spread. Tumor cells were forced to retreat in response to the aggressiveness of surrounding trophoblastic cells. Along with the co-culture time prolongation, the mouse embryo trophoblast cells continued to expand to form a clear boundary between the embryos and tumor cells (Figure 1 d, Additional file 1). After about 120 h, the area of the embryo expansion reached the maximum (Figure 1 e). The H&E staining showed that there was a blank belt between trophoblastic cells and HO8910PM cells (Figure 1 f). Most embryos began to expand when co-cultured with HO-8910 PM for about 48 h (Figure 2).

**Figure 3:**
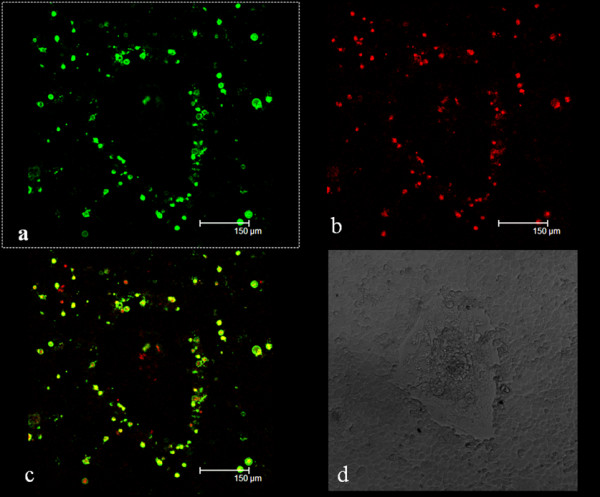
**Annexin V-EGFP/PI cell apoptosis detection.** After 96 h co-culture HO8910PM cells were stained by Annexin V-EGFP/PI and observed under laser scanning confocal microscope, **(a)** Annexin V-EGFP stained the membrane of apoptotic cells of a ring green, **(b)** PI stained the nucleoli of apoptotic or dead cells red, **(c)** dual-channel fluorescence view, **(d)** corresponding view under the light microscopy.

Annexin V-FITC/PI staining confirmed that some of the tumor cells at the peripheral of the mouse embryo underwent apoptosis when co-cultured. As shown in Figure 3, human ovarian cancer HO-8910 PM cells which contacted directly with trophoblastic cells showed cytoplasmic green or cytoplasmic green with nuclear red staining at 96 h (Figure 3 a–c).

Invasion experiment revealed that the number of transferred tumor cells was 123.1 ± 9.3 of the control group and 41.6 ± 3.0 of the experiment group. Obviously, the difference between two groups was significant. (P < 0.01)(Table 1)

**Table 1:** The effect of mouse embryos on the invasion ability of human Ovarian Cancer Cells in co-culture

**the number of transferred cells **($\bar{x}\pm s$)		
	**experiment group**	**control group**
Invasion experiment	41.6±3.0^*^	123.1±9.3

Notes: *, the result reached the extremely significant level.

**Figure 4:**
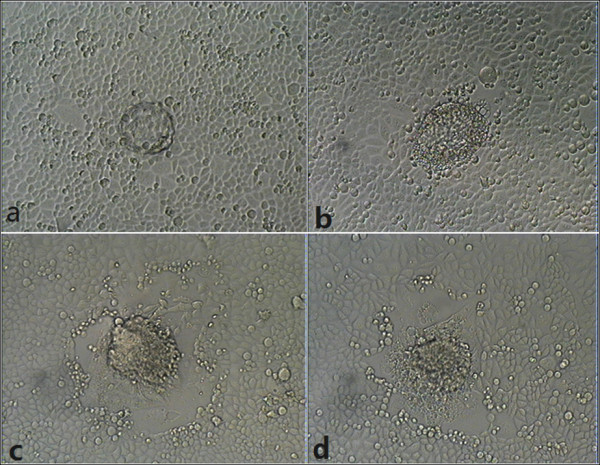
**Images of experiment group. (a)** At 24 h the mouse blastocyst adhered; **(b)** At 48 h embryonic trophoblast cells began to expand; **(c)** At 72 h tumor cells were pushed away by the growth of embryos ; **(d)** At 120 h the expansion area of trophoblast cells stopped enlarging (100 ×).

In the experiment group supplemented with MMP-9 Inhibitor I, the embryo can still adhere to human ovarian cancer cells and push away tumor cells to form their own growth space(Figure 4), exhibiting invasive behavior to tumor cells. In the control group supplemented with DMSO showed the same phenomenon. There was no significant difference between the experiment group and the control group.

## Discussion

There have been many reports about impacts of feeder cells on embryonic development in the co-culture environment. Auerbach found that different rearing environment could make embryonic cells differentiate into different type of cells which demonstrated that different culture environment influenced development of embryonic stem cells [16]. But there were few studies and reports about the effects of developmental embryos *in vitro* on feeder cells. In this study, through the co-cultured experimental model, the direct impacts of the embryo as a whole on human ovarian cancer cells HO8910PM *in vitro* were observed. The mouse blastocysts were found not only directly attached to human ovarian cancer cells which overspread the petri dish, but also inserted into the tumor cells and through further expansion of trophoblasts gradually formed their own space. Where did the tumor cells go those were already there before the blastocyst attached and expanded?

Earll L. Parr’ group found that apoptosis of epithelial cells occurred when they observed the implantation site of mice embryo in the uterus by electron microscopy [17]. But this apoptosis was orderly regulated by the body. Cucina claimed that extracts of zebrafish embryos could inhibit the proliferation of many tumor cells and induced their apoptosis [18]. In our study, after tumor cells and mouse embryos were co-cultured for some time, we found that the apoptosis of HO8910PM cells around mouse embryos increased while apoptosis of tumor cells away from mouse embryos did not increase, indicating that the impact of mouse embryos causing apoptosis of tumor cells was through direct contact between cells. Montello’s group’s study indicated that MMPs played a role during apoptosis [19], so maybe the apoptosis of HO8910PM cells was partly caused by MMPs. But MMPs cannot fully explain the reason of this phenomenon we have found, because both the embryo and tumor cells expressed MMPs as we previously found. As we know there may be species differences between human and mouse and that may cause apoptosis. But we had done a lot of work and 31 lines of cancer cells derived from different histological origins and/or with different invasiveness and metastasis were respectively co-cultured with mouse blastocysts. Mouse blastocysts invaded all kinds of cancer cells in a similar fashion. The results demonstrated that species differences were not the reason of apoptosis.

Trophoblast cells establish ways of exchanging substance with matrix during implantation by invading endometrial cells and endometrial blood vessels. This invasive behavior can be continued until mid-gestation, but it is strictly controlled both spatially and temporally [20]. The implantation process of embryo is mainly achieved through MMPs. Das found that first trimester trophoblast cells have high expression of MMP-9, and at the same time maternal decidua surrounding the blastocyst *in vivo* upregulated TIMP-3 [21,22]. Conditioned medium of decidual cells *in vitro* significantly reduced MMP-2 and MMP-9 expression of trophoblast cells but increased TIMP-1 and PAI-1 expression. These indicated that the precise dynamic balance of MMPs-TIMPs in the normal implantation course was an important mechanism of blastocyst trophoblast invading maternal decidua *in vivo*. *In vitro* co-culture environment, there was no inhibition effect of TIMPs on MMPs, which may be the explanation to why embryos display an invasive behavior to tumor cells.

In this study, during the process of co-culture, MMP-9 inhibitor (MMP-9 Inhibitor I) was added in the culture medium to inhibit the activity of MMP-9. Then the interactions of human ovarian cancer cells and embryos were observed under the microscope. The results showed that compared with the control group, the embryo was still able to adhere to human ovarian cancer cells and push away tumor cells to form their own growth space, showing the invasive behavior to tumor cells. There were no changes of invasive behavior of embryos because of the inactivation of MMP-9, indicating that MMP-9 did not play a single and independent role in this phenomenon and mouse embryos could bypass the MMP-9 way to invade tumor cells. Also maybe because when we inhibited the activity of MMP-9, meanwhile the activity of other MMPs increased such as MMP-2 by way of feedback or other ways. So we plan to use CTTHWGFTLC peptide to inhibit both the activity of MMP-9 and MMP-2 to see what will happen next.

Embryos and tumor are very similar phenomenon of life while they lead lives to two completely opposite direction. Putting the two phenomenon of life together to study and contrast may help us to understand or conquer the tumor and maybe provide a new way of thinking or open up a new path of tumor therapy.

## Competing interests

We declare that we have no competing interests.

## Authors’ contributions

XD and LF were involved in the project design, conducted the experiments and data analysis in this project and they contribute equally to the article. HZ and HQ participated in the experimental design and contributed to the statistical analysis of the data. ZW designed, coordinated and supervised the experimental studies. All authors read and approved the final manuscript.

## Supplementary Material

Additional file 1 **Dynamic image of an embryo invading human ovarian cancer cells.** The embryo and trophoblasic cells are very active. Tumor cells which contacted directly with trophoblastic cells are shrinking and collapsing and some debris is engulfed by trophoblastic cells. Click for file here
